# Identification and functional analysis of differential miRNAs in young and middle-aged adults with essential hypertension: exploring potential biomarkers and therapeutic targets

**DOI:** 10.3389/fcvm.2025.1701938

**Published:** 2026-01-30

**Authors:** Xiu-Long Niu, Xiao-Jing Wang, Xin Zhang, Wei Pang, Qiong Guo, Tao Wang, Yu-Tian Liang, Chao Liu, Fang Hao, Guo-Hong Yang, Wei Cai, Hui-Xin Li, Shao-Bo Chen, Rui Shi

**Affiliations:** 1 Department of Prevention and Therapy of Cardiovascular Diseases in Alpine Environment of Plateau, Characteristic Medical Center of PAP, Tianjin, China; 2 Tianjin Key Laboratory of Cardiovascular Remodeling and Target Organ Injury, Tianjin, China; 3 Laboratory Department, Characteristic Medical Center of PAP, Tianjin, China; 4 Medical College, Shanghai Jiao Tong University, Tianjin, China; 5 Tianjin Medical University, Tianjin, China

**Keywords:** essential hypertension, GPCPD1, miR-10b-5p, miR-744-5p, THBS2/PI3K/Akt

## Abstract

**Background:**

Essential hypertension (EH) in young and middle-aged adults is a major risk factor for cardiovascular morbidity, yet the detailed molecular mechanisms underlying this condition have not been fully understood. MicroRNAs (miRNAs) have emerged as critical regulators of gene expression and have been increasingly associated with hypertension pathogenesis.

**Methods:**

Initially, high-throughput sequencing was employed to identify miRNAs with differential expression profiles in plasma collected from six EH patients compared with six matched healthy individuals. These candidate miRNAs were then confirmed through quantitative real-time PCR (qRT-PCR). Gene Ontology (GO) and Kyoto Encyclopedia of Genes and Genomes (KEGG) analyses were conducted to elucidate the potential biological roles of these miRNAs. Mendelian randomization (MR) was subsequently applied to investigate causal associations between EH and proteins targeted by the identified miRNAs.

**Results:**

Differential expression analysis revealed 14 upregulated miRNAs and 1 downregulated miRNA in the EH group. Following this, miRNAs were enriched through MiEAA, resulting in the selection of one downregulated miRNA and four upregulated miRNAs, which were subsequently validated by qRT-PCR. KEGG pathway analysis showed that genes regulated by upregulated miRNAs were significantly enriched in pathways related to hypoxia (HIF-1 signaling pathway), inflammation (MAPK and JAK-STAT signaling pathways), vascular remodeling (Focal adhesion and TGF-beta signaling pathways), and apoptosis (p53 signaling pathway). MR analysis identified GPCPD1, regulated by hsa-miR-10b-5p, as a significant risk factor for EH (OR = 1.04, 95% CI: 1.00–1.09, *p* = 2.32 × 10^−3^). Moreover, proteins regulated by upregulated miRNAs, such as CDKN1B, PDGFRA, and THBS2, were found to be protective factors against EH.

**Conclusion:**

GPCPD1, regulated by hsa-miR-10b-5p, is a potential risk factor for EH, while CDKN1B, PDGFRA, and THBS2, regulated by upregulated miRNAs, may act as protective factors. These findings suggest new biomarkers and therapeutic targets for hypertension.

## Introduction

EH, also termed primary hypertension, remains one of the most significant global health concerns due to its widespread prevalence. Approximately 29.2% of the adult population worldwide is affected by varying stages of hypertension, a disorder that substantially raises the risk for cardiovascular complications, cerebrovascular incidents, renal impairment, and early death (1). Although pharmacological treatments and lifestyle modifications are widely accessible and effective, EH incidence continues to rise, largely driven by aging demographics, increased sedentary behaviors, poor nutritional patterns, and rising obesity rates (1). This trend presents a substantial burden on healthcare systems, particularly among young and middle-aged populations, where the onset of hypertension often marks the beginning of a cascade of health complications.

The social and economic burden of hypertension in young and middle-aged individuals is especially profound. As individuals in this age group are in their peak working years, the impact of EH extends beyond individual health, affecting productivity, quality of life, and overall economic stability. Moreover, the complications arising from untreated or poorly managed hypertension—such as stroke, heart failure, and kidney disease—further intensify healthcare costs, resulting in both direct medical expenses and indirect costs related to disability, lost wages, and early mortality. As such, addressing hypertension, particularly in young and middle-aged populations, has become a public health priority (2–4).

Despite the high disease burden and prevalence, the exact pathogenic mechanisms of EH are not yet entirely clear. The development of hypertension is known to be influenced by genetic predispositions combined with environmental exposures and lifestyle choices (5); nevertheless, the complex interactions among these factors and associated molecular networks continue to be actively explored. In recent years, significant efforts have focused on investigating genetic susceptibility to hypertension, with numerous genomic regions linked to blood pressure control identified through genome-wide association studies (GWAS) (6, 7). However, these genetic factors explain only a small fraction of the variability in blood pressure, highlighting the need to explore other molecular mechanisms that may contribute to hypertension. One such mechanism that has garnered increasing attention is the role of miRNAs.

MiRNAs are small, non-coding RNA molecules that regulate gene expression posttranscriptionally by binding directly to messenger RNA (mRNA), thereby suppressing translation or triggering mRNA degradation. They regulate diverse physiological processes such as apoptosis, cellular proliferation, differentiation, and inflammation. Increasing evidence indicates that miRNAs are involved in multiple diseases, including cancer, cardiovascular disorders, and neurological conditions. Within hypertension specifically, miRNAs appear integral to the regulation of processes essential to pathogenesis, such as endothelial integrity, vascular inflammation, smooth muscle cell proliferation, and vascular responsiveness. Recent studies propose that miRNAs not only influence blood pressure homeostasis but may also be valuable as diagnostic biomarkers and therapeutic targets for hypertension (8).

Further supporting their relevance to hypertension, miRNAs have demonstrated involvement in regulating critical signaling pathways, including endothelial dysfunction, vascular remodeling, and the renin–angiotensin–aldosterone system (RAAS) (9). Several investigations have identified aberrantly expressed miRNAs in hypertensive subjects, underscoring their potential role in hypertension development. Nevertheless, the specific miRNAs most influential in driving primary hypertension, especially among young and middle-aged adults who represent a particularly vulnerable population, require additional elucidation.

Given the increasing prevalence of EH, particularly in young and middle-aged populations, and the potential role of miRNAs in regulating key pathways involved in hypertension, there is an urgent need for further research in this area. Understanding the specific miRNAs that regulate hypertension-related pathways could lead to novel diagnostic tools, as well as targeted therapies that extend beyond traditional pharmacological treatments. The current study aims to fill this gap by identifying differentially expressed miRNAs in a cohort of young and middle-aged EH patients and exploring their regulatory effects on target genes involved in hypertension. By investigating these miRNAs, their target proteins, and associated signaling pathways, we hope to provide new insights into the molecular mechanisms driving primary hypertension and uncover potential biomarkers and therapeutic targets for better management of this prevalent condition.

## Materials and methods

### Study design

Our study recruited a group of young and middle-aged EH patients (*n* = 50) alongside 14 normotensive healthy individuals (NBP group). Initially, blinded RNA sequencing was performed to analyze circulating miRNAs obtained from blood samples. Following sequencing and quantification, miRNAs demonstrating differential expression were identified. To elucidate potential functional significance, functional enrichment was assessed using miEAA, resulting in the identification of five significantly dysregulated miRNAs related to hypertension, which were subsequently validated through RT-PCR assays. Thereafter, predictive target genes of these miRNAs underwent further analysis using GO annotation (including biological processes), KEGG pathway analysis, and protein–protein interaction (PPI) network construction. Finally, we used MR to conduct causal analysis between key proteins and EH. This approach identified hypertension-related pathways and key proteins in young and middle-aged individuals, uncovering novel biomarkers for EH in this population.

### Objects

This study included middle-aged patients with EH (EH group, *n* = 50) and controls (NBP group, *n* = 14), all diagnosed at the Department of Cardiovascular Diseases, Characteristic Medical Center of PAP, between April and December 2023. Plasma samples from six selected subjects (four males and two females) were used for miRNA high-throughput next-generation sequencing. Inclusion criteria were as follows: Participants' blood pressure status was classified according to the 2018 Guidelines for the Prevention and Treatment of Hypertension in China. Systolic blood pressure (SBP) was measured three times consecutively on the same day, without prior use of antihypertensive drugs. Individuals with SBP ≥ 140 mmHg and/or diastolic blood pressure (DBP) ≥ 90 mmHg were categorized as hypertensive. Isolated systolic hypertension referred specifically to those exhibiting SBP ≥ 140 mmHg but DBP <90 mmHg. Hypertensive patients currently on antihypertensive drugs but with blood pressure readings <140/90 mmHg were also included. Participants were excluded if they had taken antihypertensive medications; had secondary hypertension, white coat hypertension, coronary atherosclerotic heart disease, heart failure, severe arrhythmias (such as atrial fibrillation), cerebrovascular diseases, diabetes, severe organ dysfunction (heart, liver, kidney), metabolic disorders (e.g., hyperthyroidism), peripheral vascular disease, cor pulmonale, rheumatological, or hematologic diseases; or had used myeloproliferative drugs. Participants who had incomplete clinical records or had experienced infectious illnesses in the preceding 2 weeks were also excluded from the study. Ethical approval was granted by the Institutional Ethics Committee, and all subjects provided written informed consent prior to participation.

### Clinical data collection

Clinical parameters included age, sex, body mass index (BMI), smoking habits (defined as daily cigarette consumption exceeding one cigarette for no less than 6 months), alcohol intake patterns (defined as weekly alcohol consumption for at least 6 months), familial hypertension history (defined as EH diagnosis in at least one family member within three generations), and historical medication usage.

### Blood pressure measurement

Participants rested quietly for no less than 5 min prior to blood pressure assessment, abstained from smoking and caffeine intake for at least 30 min beforehand, and emptied their bladders. Measurements were carried out with subjects seated comfortably, their arms supported at heart level. An Omron electronic sphygmomanometer, meeting standards set by the Association for the Advancement of Medical Instrumentation (AAMI), was used for upper-arm blood pressure determination. A properly sized cuff (standard dimensions: length 22–26 cm, width 12 cm; or 32 cm length for larger arm circumferences) was applied. Initial measurements were performed on both arms, and subsequent readings utilized the arm with the higher initial blood pressure. Two measurements separated by an interval of 1–2 min were averaged. If the difference in SBP or DBP exceeded 5 mmHg, a third reading was taken, and the average of all three was recorded.

### RNA sequencing data preprocessing

Raw sequencing outputs commonly include adapter sequences, low-quality bases, and terminal artifacts, negatively affecting downstream analysis. Therefore, initial data processing was essential for generating high-quality datasets. Filtering was conducted using the fastx toolkit (version 0.0.13) according to the following criteria: (1) adapter removal, (2) elimination of bases from the 3′ ends exhibiting low quality [*Q*-score <10, where *Q* = −10 log(error ratio)], and (3) discarding sequences shorter than 10 nucleotides.

### Classification and annotation of small RNAs

Filtered clean reads, 18–44 nucleotides in length, were mapped onto the reference genome (version: rn6_ensembl) retrieved from Ensembl FTP, using the Bowtie alignment tool with a permissible mismatch of one base. MiRNAs and other classes of small RNAs were annotated according to miRNA data from miRBase and additional non-coding RNA information available in the reference genome database (10).

### Quantitative analysis of miRNA expression

To reliably compare miRNA expression across samples, mapped read counts were normalized employing the trimmed mean of *M*-values (TMM) normalization method and then converted into expression levels reported as transcripts per million (TPM), calculated as miRNA read counts × 10^6^/total mapped reads. Biological replicates were included to strengthen experimental reproducibility and reliability. Correlations among sample expression profiles were examined using principal component analysis (PCA), a statistical technique reducing data dimensionality through linear recombination into principal components, facilitating the identification of outliers and sample clustering (11).

### Differential expression analysis of miRNAs

Differential expression analysis of miRNAs across groups was performed using edgeR software. Raw *p*-values were adjusted for multiple hypothesis testing using false discovery rate (FDR) correction, resulting in *q*-values. Fold changes in miRNA expression were computed based on TPM values. MiRNAs were considered significantly differentially expressed if they met the criteria: (1) *p* ≤ 0.05 and (2) log2 fold change ≥ 1.3. Visualization of significant miRNAs was conducted using volcano plots created by the ggplot2 package in R, with clear annotation of miRNAs meeting significance criteria.

### miRNA enrichment analysis

Disease-related enrichment analysis was conducted on significantly differentially expressed miRNAs using the online tool miEAA. Through this analysis, miRNAs specifically implicated in hypertension were identified for further validation and functional exploration.

### qRT-PCR validation

Expression levels of candidate miRNAs identified through sequencing were validated using qRT-PCR. Plasma miRNA extraction was carried out using the miRNA extraction kit (CW0627S; Kangwei Biotechnology Co.) following provided protocols. Reverse transcription was executed by mixing 3.75 µL total RNA, 5 µL of 2× mRQ Buffer, and 1.25 µL mRQ Enzyme in a 200-µL reaction tube and incubating (37°C for 1 h; 85°C for 5 min). The reaction was subsequently diluted with 90 µL ddH2O, achieving a final volume of 100 µL. Amplification was performed utilizing SYBR® Premix Ex Taq™ II (Tli RNaseH Plus) according to the manufacturer’s instructions. Quantification of miRNA expression was done with the ABI QDX fluorescence quantitative PCR system, and relative expression was calculated via the 2^−ΔΔCt^ method. Primer sequences were synthesized by Shanghai Shenggong Biotechnology Co.

### Exploration of target genes for differentially expressed miRNAs

Target genes regulated by identified differentially expressed miRNAs were investigated to further clarify their roles in disease mechanisms. Using the multiMiR R package, potential targets were identified according to stringent selection criteria: (1) confirmed interactions by luciferase reporter assays or cross-linking immunoprecipitation (CLIP) and (2) inclusion in both the miRTarBase and TarBase databases.

### Enrichment analysis

To elucidate the biological implications and cellular mechanisms related to the identified miRNAs, GO and KEGG pathway enrichment analyses were conducted using the clusterProfiler R package. These analyses investigated significant enrichment of biological pathways or functions among selected genes, with results adjusted by FDR for multiple comparisons. STRING (version 11.5) was applied to explore PPI networks, clustering target genes based on text-mining methods. Networks were visualized using Cytoscape (version 3.9.0) software.

### MR estimates

Plasma protein measurements were obtained from the deCODE database, while GWAS summary statistics for EH were accessed from the UK Biobank (UKBB). Multiple MR approaches—particularly inverse variance weighting (IVW) for multi-SNP analyses and the Wald ratio for single-SNP scenarios—were applied to assess causal relationships between the identified target proteins and EH.

### Statistical analysis

Clinical characteristics—including gender, age, BMI, smoking status, familial hypertension, and blood pressure values—were statistically evaluated using multivariate regression analysis via the compareGroups R package. Statistical significance was determined by *p*-values less than 0.05.

## Results

### General information

Supplementary Table 1 presents the baseline characteristics, indicating an approximate mean age of 40 years for both EH patients and control participants. Statistical analyses showed no marked differences between groups in age, gender ratio, smoking habits, heart rate, or serum creatinine. However, significant disparities were evident for BMI, uric acid, and lipoprotein profiles, which are established risk factors linked to EH among young and middle-aged adults.

### RNA sequencing profiling of circulating miRNA signature

After comprehensive quality evaluation, six plasma samples per group were selected for RNA sequencing. PCA results are depicted in Supplementary Figure 1A. Within plasma samples, sequences ranging from 18 to 40 nucleotides were identified as small non-coding RNA (sncRNA) transcripts. Exploratory analysis revealed that approximately 32% corresponded to sncRNAs, while approximately 11% mapped to other recognized non-coding RNAs (ncRNAs) in the human genome. The majority (69.36%) remained unannotated, whereas only 1% of sequences were unmapped but similar in size to sncRNAs. Subsequent bioinformatics processing identified a total of 13,893 miRNAs (Supplementary Figure 1B).

### Differential miRNA expression

**Figure 1:**
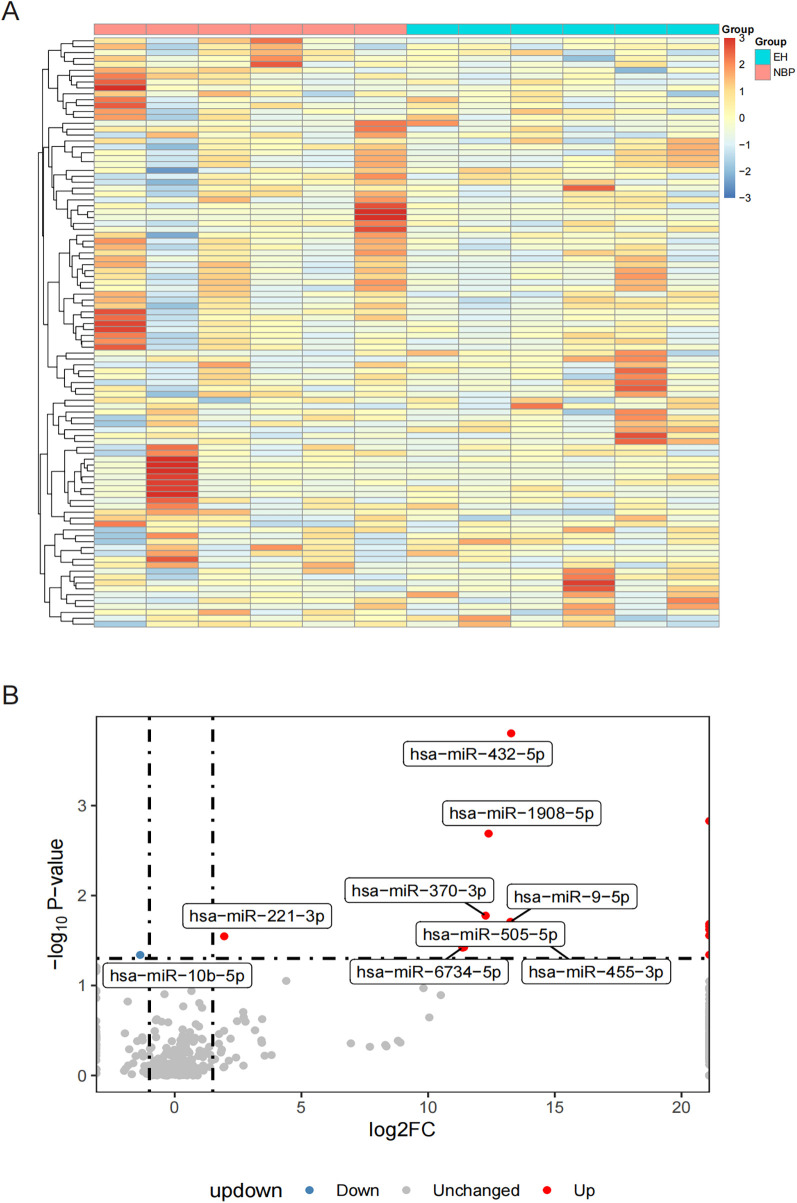
Heatmap and volcano plots illustrating differential miRNA expression. **(A)** Heatmap presenting the expression patterns of the 100 most significantly expressed miRNAs across 12 samples divided into two groups; and **(B)** volcano plot demonstrating differentially expressed miRNAs, with red dots representing significantly increased miRNAs, blue dots indicating significantly decreased miRNAs, and gray dots denoting miRNAs lacking significant expression differences.

Figure 1A presents a heatmap of the top 100 most highly expressed miRNAs from sequencing results. Differential expression analysis revealed that, compared to the NBP group, the EH group had 14 upregulated miRNAs and 1 downregulated miRNA (Supplementary Tables 2 and 3). Figure 1B shows the upregulated miRNAs, which include hsa-miR-432-5p, hsa-miR-411-5p, hsa-miR-1908-5p, hsa-miR-370-3p, hsa-miR-9-5p, hsa-miR-744-5p, hsa-miR-26b-3p, hsa-miR-4433b-3p, hsa-miR-3679-5p, hsa-miR-221-3p, hsa-miR-455-3p, hsa-miR-505-5p, hsa-miR-6734-5p, and hsa-miR-342-5p. The only significantly downregulated miRNA was hsa-miR-10b-5p. For more accurate analysis, we performed disease enrichment screening of the significantly differentially expressed miRNAs using MiEAA (Supplementary Table 4). Five miRNAs were found to be significantly associated with hypertension. Among them, hsa-miR-10b-5p was significantly downregulated in EH (log2Fold = 1.36; *p* = 4.57 × 10^−2^). The other four miRNAs—hsa-miR-370-3p (log2Fold = 12.26; *p* = 1.67 × 10^−2^), hsa-miR-9-5p (log2Fold = 13.24; *p* = 1.96 × 10^−2^), hsa-miR-744-5p (log2Fold = Inf; *p* = 2.07 × 10^−2^), and hsa-miR-221-3p (log2Fold = 1.95; *p* = 2.84 × 10^−2^)—were significantly upregulated in EH. These five differentially expressed miRNAs were selected for further analysis in the subsequent studies.

### miRNA qPCR validation

**Figure 2:**
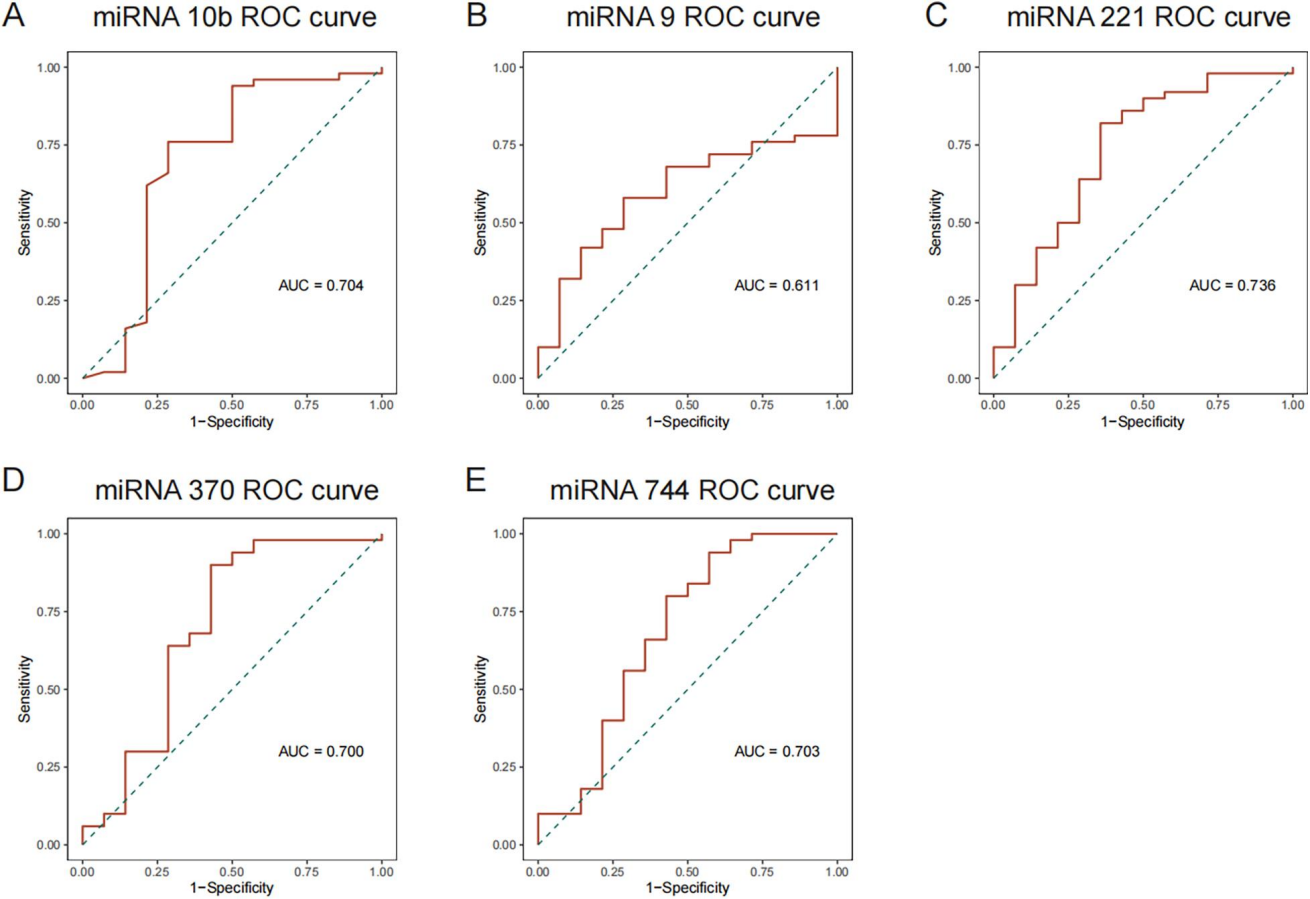
ROC curve analyses for differentially expressed miRNAs. **(A)** ROC curves for downregulated miRNAs; and **(B–E)** ROC curves for upregulated miRNAs.

To verify RNA sequencing outcomes, qPCR was conducted on plasma samples from 50 EH patients and 14 control subjects. Compared to the controls, EH participants exhibited significant reductions in hsa-miR-10b-5p levels and significant elevations in hsa-miR-221-3p, hsa-miR-370-3p, and hsa-miR-744-5p (all *p* < 0.01). While hsa-miR-9-5p exhibited an increasing trend, this difference lacked statistical significance (Supplementary Table 6). ROC curve analysis derived from qPCR results (Figure 2) revealed that three miRNAs (hsa-miR-221-3p, AUC = 0.736; hsa-miR-370-3p, AUC = 0.700; hsa-miR-744-5p, AUC = 0.703) demonstrated satisfactory discriminative performance (AUC > 0.7). Conversely, hsa-miR-10b-5p and hsa-miR-9-5p presented lower discriminatory capabilities (AUC = 0.611). Overall, these qPCR data aligned closely with sequencing and enrichment analyses, reinforcing the significance of these five miRNAs in young and middle-aged EH pathology.

### Differential miRNA target genes

Further exploration of biological mechanisms regulated by these validated miRNAs was performed by identifying their target genes using the multiMiR R package. Supplementary Table 7 indicates that among the upregulated miRNAs, hsa-miR-221-3p demonstrated the strongest regulatory potential, with 1,770 target genes, followed by hsa-miR-9-5p (996 targets), hsa-miR-744-5p (689 targets), and hsa-miR-370-3p (82 targets). Among the downregulated miRNAs, hsa-miR-10b-5p regulated 380 target genes (Supplementary Table 8). Accordingly, subsequent analyses focused primarily on hsa-miR-221-3p, hsa-miR-9-5p (upregulated), and hsa-miR-10b-5p (downregulated).

### Enrichment analysis of differentially expressed miRNAs

**Figure 3:**
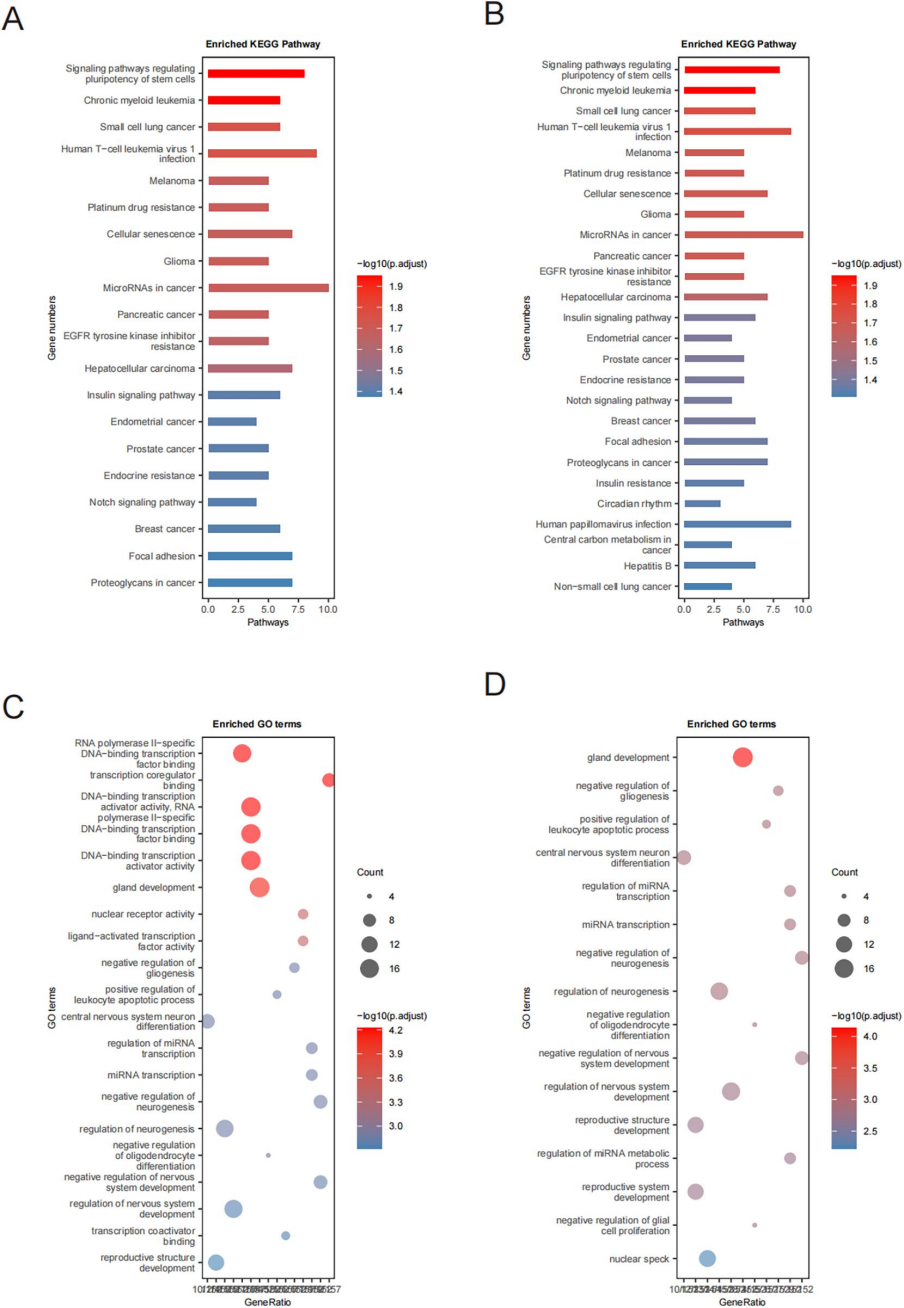
KEGG and GO enrichment analyses. **(A,B)** KEGG pathway enrichment analysis of proteins targeted by miRNAs showing upregulated and downregulated expression, respectively; and **(C,D)** GO enrichment analysis of proteins targeted by miRNAs showing upregulated and downregulated expression, respectively.

To elucidate the potential biological processes and pathways regulated by these miRNAs, enrichment analyses (KEGG and GO) were performed using the clusterProfiler R package. For genes targeted by upregulated miRNAs, KEGG enrichment prominently identified hypoxia-related pathways, notably the HIF-1 signaling pathway (*p*.adjust = 2.00 × 10^−4^) and the Insulin signaling pathway (*p*.adjust = 1.98 × 10^−4^). In addition, pathways linked to vesicular trafficking (endocytosis and regulation of actin cytoskeleton), inflammation (MAPK and JAK-STAT signaling pathways), vascular remodeling (focal adhesion, TGF-beta signaling, and apelin signaling pathways), and apoptosis (p53 signaling and apoptosis) were significantly enriched (Supplementary Table 10, Figure 3A).

For the downregulated miRNAs, enrichment analyses highlighted significant involvement of pathways such as notch signaling, focal adhesion, and insulin resistance (Supplementary Table 9, Figure 3B). These findings suggest critical regulatory roles for these miRNAs in cellular differentiation, metabolic adjustments, inflammation control, and vascular structural changes, which collectively modulate cardiovascular homeostasis and stress responses.

GO enrichment analysis demonstrated that target genes of downregulated miRNAs were predominantly enriched in biological processes associated with central nervous system neuron differentiation, negative regulation of neurogenesis, and regulation of miRNA metabolic processes (Figure 3D), indicating their importance in neural development and function. Conversely, targets of upregulated miRNAs were mainly implicated in processes such as DNA-binding transcription activator activity, nuclear receptor signaling, mitochondrial apoptotic regulation, and cell cycle progression, highlighting their relevance to cell viability and metabolism (Figure 3C). In addition, an association with cancer-related signaling pathways suggested possible connections between these miRNAs and oncogenic mechanisms (Figure 3, Supplementary Table 11).

In summary, the observed upregulated miRNAs primarily influenced hypoxic responses, inflammatory reactions, apoptotic signaling, and vascular remodeling, prominently involving HIF-1, insulin, and MAPK pathways. In contrast, downregulated miRNAs notably participated in pathways including notch signaling, focal adhesion, and insulin resistance, emphasizing their roles in cellular differentiation and metabolic regulation. Collectively, these miRNAs and associated pathways provide meaningful insights into the molecular mechanisms underlying hypertension and related cardiovascular complications among young and middle-aged individuals.

### PPI network analysis

**Figure 4:**
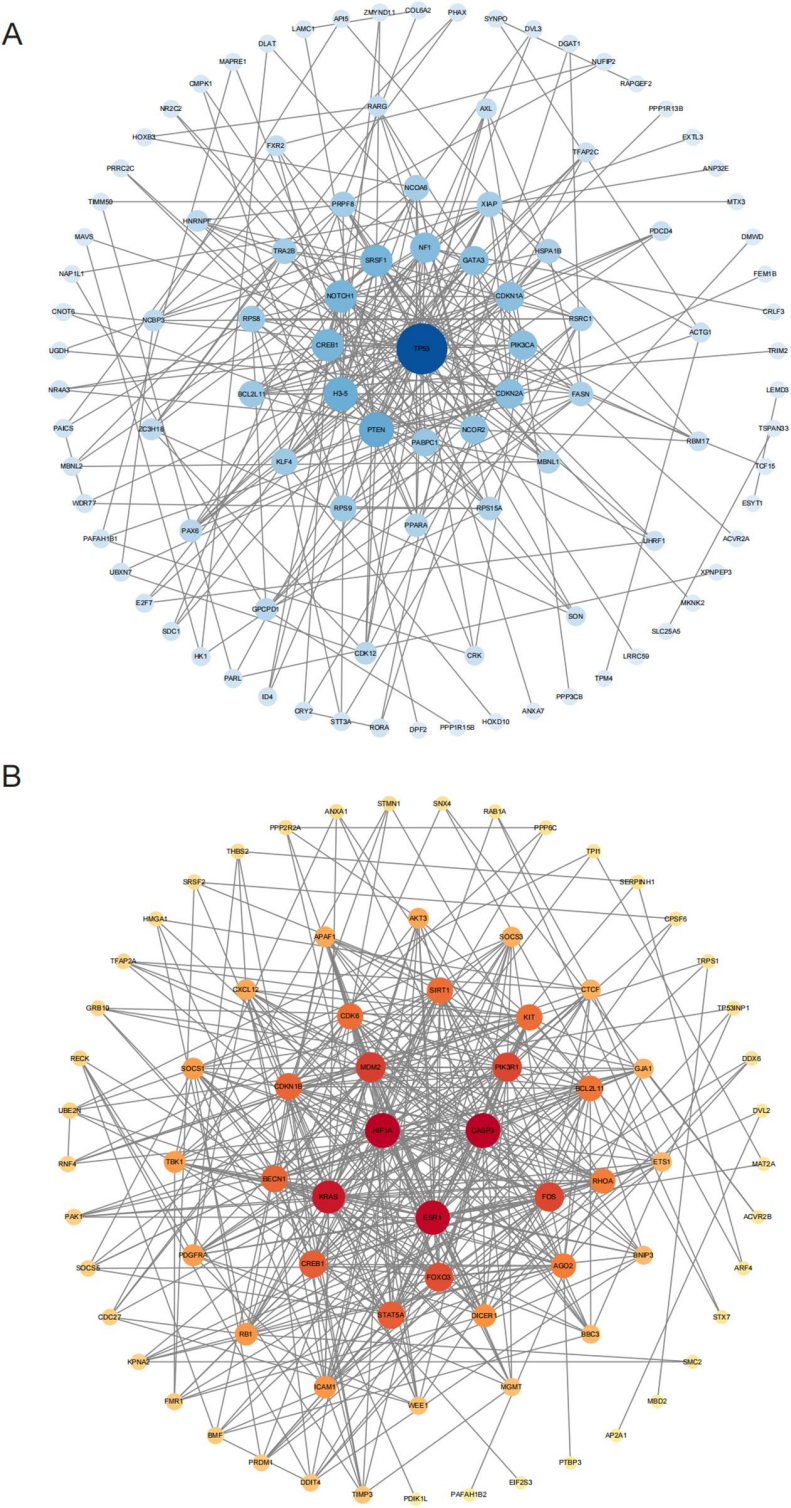
PPI network analyses for targets of differentially expressed miRNAs. **(A)** PPI network for proteins targeted by downregulated miRNAs; and **(B)** PPI network for proteins targeted by upregulated miRNAs. Gray boxes depict original STRING-generated results, while node size and color depth indicate each protein's significance within the interaction network.

We performed a PPI network analysis on genes targeted by the identified miRNAs to better characterize the molecular interactions underlying hypertension. Among targets of the upregulated miRNAs, HIF1 exhibited the most prominent degree of connectivity, followed by ESR1 and CASP3 (Figure 4B). These proteins are critically involved in key cellular processes such as the hypoxic response, apoptosis, and cell survival. Their strong interconnections highlight their essential roles within the regulatory pathways influenced by elevated miRNAs, potentially contributing to vascular remodeling, inflammation, and apoptosis observed in young and middle-aged EH patients.

In contrast, downregulated miRNAs primarily targeted TP53 and SNCOR2. TP53 is a well-established tumor suppressor protein regulating cell cycle control, DNA repair mechanisms, and apoptosis. Its involvement suggests a role in controlling apoptosis and proliferation of vascular smooth muscle cells, which are crucial events in hypertension pathogenesis. SNCOR2 is involved in metabolic regulation and may impact endothelial function and vascular integrity. These findings indicate that TP53 and SNCOR2 are crucial for the pathways regulated by downregulated miRNAs, and their modulation may be central to maintaining vascular integrity and metabolic balance in the context of high blood pressure.

In conclusion, the identification of key proteins such as HIF1, ESR1, TP53, and SNCOR2 through PPI analysis emphasizes their potential impact on the pathogenesis of young and middle-aged EH. By modulating these protein targets, differentially expressed miRNAs may significantly affect essential cellular mechanisms, including inflammation, apoptosis, and vascular remodeling, thereby influencing hypertension development. These findings provide valuable insights into the molecular pathogenesis of hypertension and highlight potential biomarkers and therapeutic candidates for clinical management.

### MR analysis

We conducted a causal analysis using MR to assess the relationship between plasma proteins and differentially expressed miRNAs. Plasma protein data from the deCODE database were used to identify proteins regulated by differential miRNAs. A total of 141 plasma proteins regulated by upregulated miRNAs (Supplementary Table 11) and 15 plasma proteins regulated by downregulated miRNAs (Supplementary Table 12) were selected for MR analysis in relation to young and middle-aged EH.

**Figure 5:**
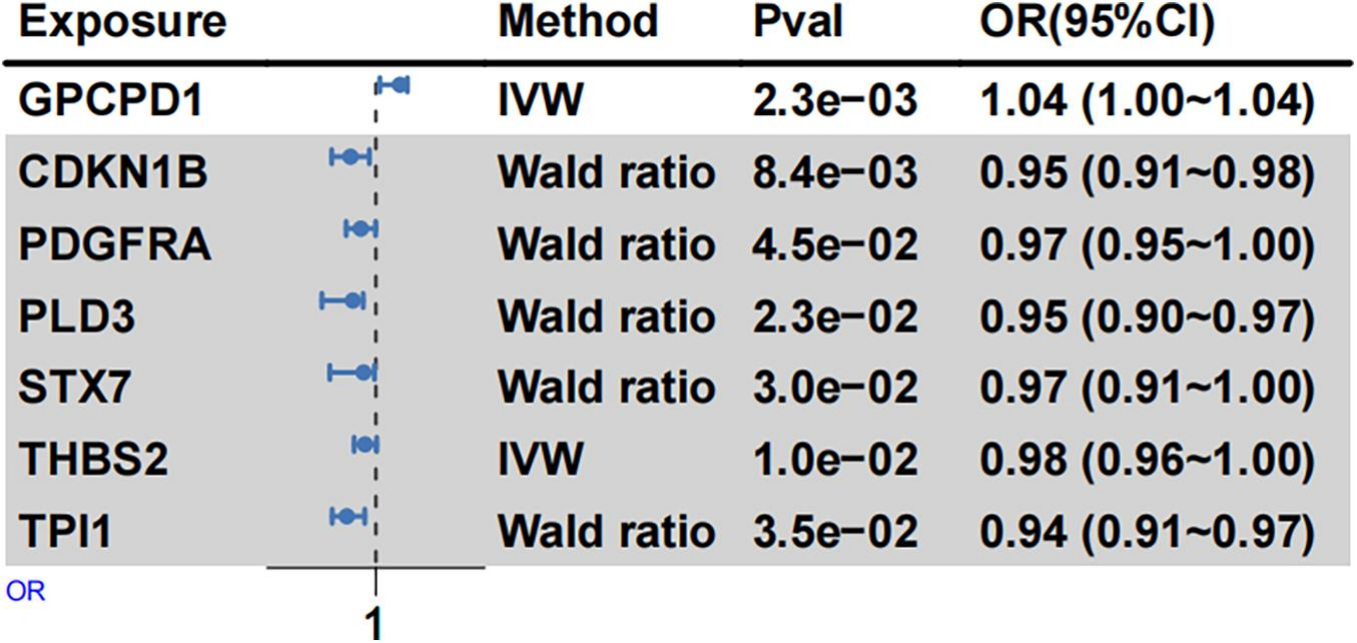
MR analysis summarizing protein associations with EH risk. Forest plots depict causal associations and their corresponding effect sizes between selected proteins (from deCODE) and EH risk. Odds ratios (ORs) were calculated using fixed-effect IVW and Wald ratio methods. Squares represent OR estimates, and horizontal lines reflect their 95% confidence intervals (CIs).

MR analysis identified a significant causal link between genetically predicted plasma levels of GPCPD1 and increased EH susceptibility among young and middle-aged individuals (IVW: OR = 1.04, 95% CI: 1.00–1.09, *p* = 2.32 × 10^−3^) (Figure 5, Supplementary Table 14). GPCPD1, targeted by downregulated hsa-miR-10b, thus appears to be a risk-associated factor for EH. Conversely, genetically determined plasma levels of CDKN1B (IVW: OR = 0.95, *p* = 8.36 × 10^−3^) and PDGFRA (IVW: OR = 0.97, *p* = 4.49 × 10^−2^), both regulated by hsa-miR-221-3p, exhibited significant inverse associations with EH risk (Figure 5, Supplementary Table 13). Similarly, PLD3 and THBS2, regulated by hsa-miR-9-5p and hsa-miR-744-5p, respectively, demonstrated negative causal associations with EH. Furthermore, STX7 and TPI1, targeted by hsa-miR-221-3p and hsa-miR-9-5p, also showed negative causal associations with young and middle-aged EH risk. These results suggest that these proteins, regulated by upregulated miRNAs, may serve as protective factors against young and middle-aged EH.

## Discussion

Young and middle-aged EH is a multifactorial disorder that involves complex interactions between genetic, environmental, and molecular factors (12, 13). Although significant progress has been made in identifying risk factors and genetic variants, the precise molecular mechanisms underlying hypertension remain unclear (14). MiRNAs have been increasingly recognized as crucial regulators of gene expression, influencing several vital biological processes such as apoptosis, inflammatory responses, and vascular remodeling, all central to EH pathophysiology (15). Our study explored the specific contributions of differentially expressed miRNAs, their target genes, and the corresponding signaling pathways in the pathogenesis of EH.

Among the significantly upregulated miRNAs identified in our analysis, hsa-miR-221-3p has previously been studied extensively in cardiovascular contexts, particularly for its role in endothelial cell migration and proliferation (16–18). Elevated expression of hsa-miR-221-3p may lead to increased vascular smooth muscle cell proliferation and impaired endothelial function, thus exacerbating hypertensive vascular remodeling. Previous studies indicate that hsa-miR-221-3p directly suppresses CDKN1B (19), a known cyclin-dependent kinase inhibitor that regulates cell cycle progression (20). Through repression of CDKN1B, hsa-miR-221-3p might enhance proliferation of endothelial and vascular smooth muscle cells, contributing to structural remodeling associated with hypertension. Moreover, hsa-miR-221-3p targets PDGFRA, a receptor for platelet-derived growth factor critically involved in atherosclerosis and vascular fibrosis (21, 22). Collectively, these findings strengthen the evidence supporting the role of hsa-miR-221-3p in EH development via modulation of pathways that promote vascular remodeling and structural changes within blood vessels (23).

Hsa-miR-9-5p was another upregulated miRNA found in the EH group and has been implicated in inflammation and apoptosis, two key processes in the pathogenesis of EH. The miRNA hsa-miR-9-5p has been shown to regulate inflammatory pathways (24), especially the MAPK signaling pathway (25), which is known to mediate responses to stress and inflammation in vascular cells. In our study, hsa-miR-9-5p targeted PLD3 (26), a protein involved in phospholipid metabolism (27), and THBS2 (28), which stabilizes the extracellular matrix in vascular tissues. Upregulation of hsa-miR-9-5p may promote endothelial dysfunction by increasing inflammation and altering the balance of extracellular matrix components. This is consistent with previous findings that hsa-miR-9-5p promotes a pro-inflammatory state in vascular endothelial cells and smooth muscle cells, a hallmark of hypertension and atherosclerosis.

Moreover, hsa-miR-9-5p also targets CASP3, an essential protein involved in apoptosis (29). Hypertension has long been associated with increased vascular cell apoptosis, contributing to the loss of endothelial cells and the thickening of the vascular wall. By regulating CASP3, hsa-miR-9-5p could influence vascular cell survival, further contributing to the vascular remodeling observed in EH.

Hsa-miR-370-3p and hsa-miR-744-5p were also markedly increased in EH, indicating their potential importance in vascular remodeling processes. Specifically, hsa-miR-370-3p influences key signaling cascades such as TGF-beta, a pathway crucially involved in extracellular matrix synthesis and vascular smooth muscle cell activity (30). Given the established role of TGF-beta signaling in hypertensive vascular fibrosis, hsa-miR-370-3p may enhance vascular stiffness, a significant characteristic of EH, by modulating this pathway (31).

Similarly, hsa-miR-744-5p affects pathways related to inflammation and cell survival (32). It has been suggested that hsa-miR-744-5p may exacerbate endothelial dysfunction by modulating inflammatory responses, which are crucial for the initiation and progression of hypertension. The miRNA's influence on apoptosis signaling further supports its role in vascular remodeling and the loss of endothelial cell integrity in EH.

The downregulated miRNA, hsa-miR-10b-5p, plays a critical role in lipid metabolism through the regulation of GPCPD1, a key enzyme involved in glycerophospholipid metabolism (19). GPCPD1 is essential for maintaining cell membrane integrity, and dysregulation of lipid metabolism is commonly observed in hypertension. The downregulation of hsa-miR-10b-5p in EH could lead to an increase in GPCPD1, disrupting lipid homeostasis in vascular cells and contributing to endothelial dysfunction, a major feature of hypertension. This provides new insights into how altered lipid metabolism might be linked to the vascular changes observed in hypertensive individuals.

Our PPI network analysis revealed several critical proteins, including TP53, SNCOR2, HIF1, ESR1, and CASP3, with high interaction scores and involvement in key regulatory processes such as apoptosis, stress response, and metabolism. Although these proteins did not appear in our MR results, the absence of suitable instrumental variables for these proteins explains their exclusion from the analysis, rather than indicating a contradiction with the results. TP53, in particular, is known to regulate apoptosis and cellular stress responses, both of which are critical in maintaining vascular health (33). Similarly, HIF1 and ESR1 regulate key responses to hypoxia and estrogen, which influence vascular tone and endothelial function (34, 35).

Our observations support earlier research highlighting miRNAs as important mediators in hypertension-related mechanisms, particularly those governing vascular remodeling, apoptosis, and inflammatory responses. Notably, miRNAs upregulated in our study, including hsa-miR-221-3p and hsa-miR-9-5p, likely promote inflammation and structural alterations in blood vessels, both central elements of EH pathology. Conversely, decreased expression of hsa-miR-10b-5p sheds new light on the connection between lipid metabolism and hypertension. These findings underscore the potential utility of miRNAs as biomarkers for early detection and as therapeutic targets in EH treatment. Moreover, the identification of these miRNAs and their specific target genes provides opportunities to explore innovative miRNA-based therapeutic interventions, such as miRNA mimics or inhibitors, to regulate gene expression critical to EH pathogenesis.

## Conclusion

In conclusion, our study's identification of differentially expressed miRNAs and their associated gene targets enhances the current understanding of molecular mechanisms underpinning EH in young and middle-aged populations. Therapeutic targeting of these miRNAs may offer novel avenues for more effective clinical management and preventive strategies for hypertension.

## Data Availability

The original contributions presented in the study are included in the article/supplementary material, further inquiries can be directed to the corresponding author(s).
