# Supplementary material for: Identification and functional analysis of differential miRNAs in young and middle-aged adults with essential hypertension: exploring potential biomarkers and therapeutic targets
Source: Front Cardiovasc Med. 2026 Jan 30;12:1701938. doi: 10.3389/fcvm.2025.1701938 (PMC12901467; doi:10.3389/fcvm.2025.1701938)
Supplement: Supplementary file 2 [file Image1.pdf]

A B

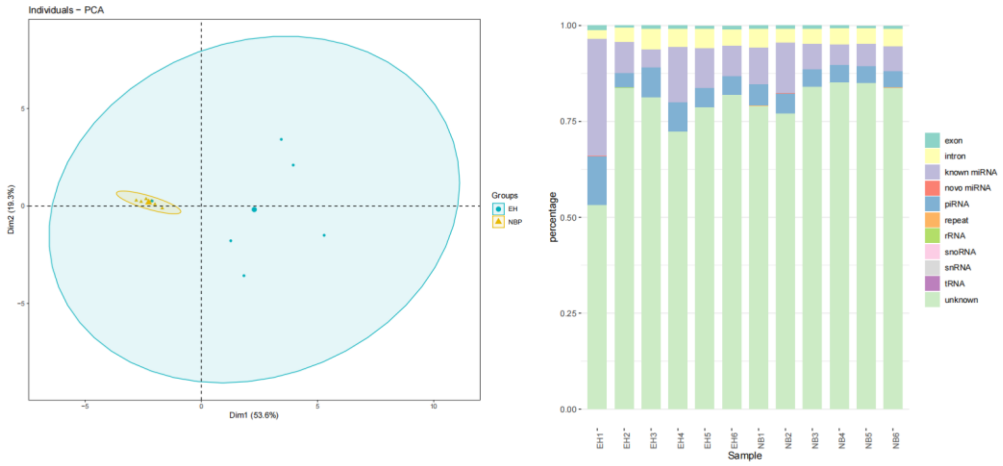

Figure S1. Data Quality Control Across Two Sample Groups.

A, Principal component analysis (PCA) diagram. B, Proportion of different types of small RNA
